# Supplementary figures and images for: Effect of Chitosan-Diosgenin Combination on Wound Healing
Source: Int J Mol Sci. 2023 Mar 6;24(5):5049. doi: 10.3390/ijms24055049 (PMC10003508; doi:10.3390/ijms24055049)

## Supplementary Materials

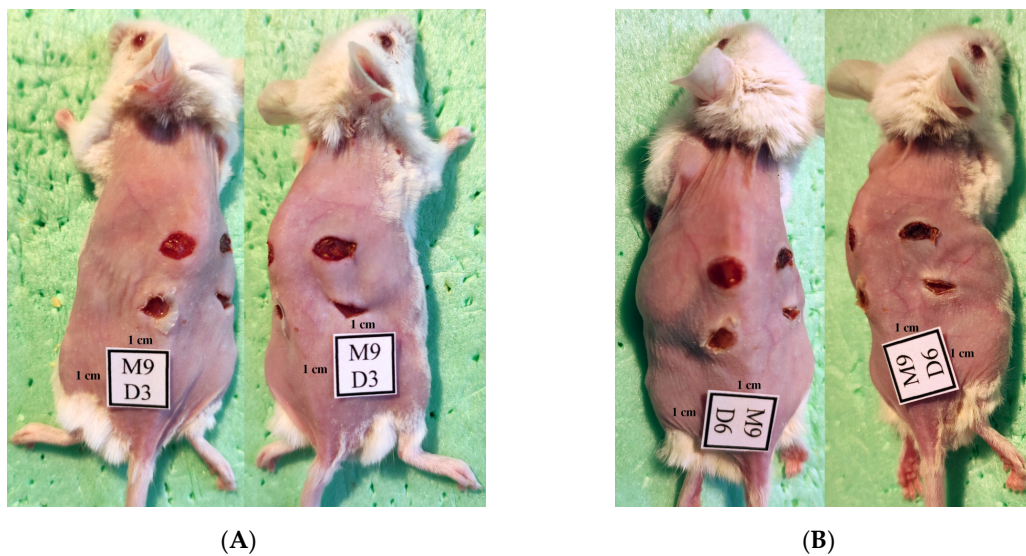

**Figure S1.** Digital imaging of the wound areas after D3 (A) and D6 (B) treatment.

Supplement: Supplementary file 1 [file ijms-24-05049-s001.zip › ijms-2219667-supplementary.pdf]
